# Improve the Efficiency of Surgery for Femoral Shaft Fractures with A Novel Instrument: A Randomized Controlled Trial

**DOI:** 10.1371/journal.pone.0154332

**Published:** 2016-04-26

**Authors:** Haitao Xu, Wenjing Yin, Peichun Hsu, Hui Qin, Zhiquan An, Changqing Zhang, Jiagen Sheng

**Affiliations:** Department of Orthopedics, Shanghai Jiao Tong University Affiliated Sixth People’s Hospital, Shanghai, China; Harvard Medical School/BIDMC, UNITED STATES

## Abstract

**Objective:**

To improve the efficacy of closed reduction and wire guiding during intramedullary nail internal fixation in femoral shaft fractures.

**Methods:**

A novel instrument was designed and manufactured. Sixty-eight patients were enrolled from February 2011 to December 2013. The instrument designed was used during the operation in the experimental group, but not in the control group.

**Results:**

All patients exhibited fracture union, excluding 1 patient in the experimental group and 2 in the control group who had non-union; all of whom achieved fracture union with reoperation. There were no statistically significant differences in operative blood loss or duration of hospital stay between the groups (P > 0.05). The operative time, frequency of wire drilling, and number of open reduction cases, were significantly smaller in the experimental group than in the control group (P < 0.05).

**Conclusion:**

Femoral shaft fractures are difficult to reduce using general methods; the novel instrument showed high clinical value and proved effective and safe in assisting with closed reduction and intramedullary nail fixation for femoral shaft fractures.

**Trial Registration:**

ChiCTR ChiCTR-ICR-15007335

## Introduction

Femoral shaft fractures are very common in orthopedic emergencies. Options for the treatment of femoral fractures have become increasingly available, and have been proven effective. In recent years, closed reduction and interlocking intramedullary nail internal fixation has become the standard treatment for femoral shaft fractures[1–5]. During the operation, 2 issues affect the operative process and time. Firstly, fracture reduction depends primarily on traction, and substantial time is required in order to obtain a satisfactory reduction. Second, it is not easy to determine the entry point and entry direction into the proximal medullary cavity for the guidewire, as the normal anatomic structure has often changed with fracture displacement. Currently, there remains no convenient and effective instrument assisting with fracture reduction and the wire-guiding process. Surgeons depend primarily on their experience in performing reduction and guidewire insertion[6]. To make the operation easier and more convenient to conduct, we have manufactured a novel instrument that improves the efficiency of the operation. From February 2011 to December 2013, we undertook a prospective randomized study to evaluate the efficacy of instrument presented, for use in intramedullary nail internal fixation, compared with traditional methods for treatment of femoral shaft fractures.

## Materials and Methods

### 1 Patients

**Fig 1:**
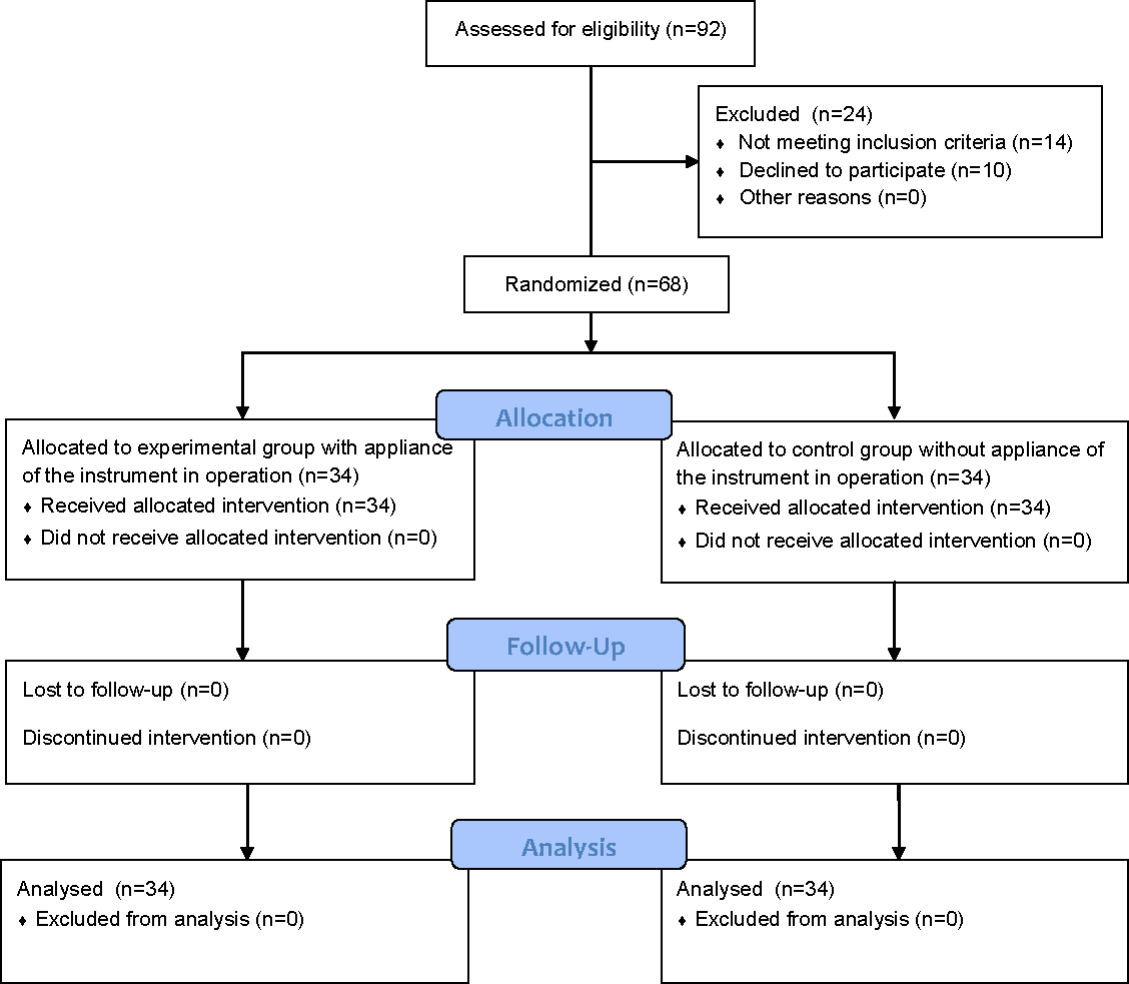
CONSORT flow diagram. Flow diagram displaying the progress of all participants throughout the trial.

Potential participants of this trial were recruited between February 2011 and December 2013 from Shanghai Sixth People’s Hospital. Patients were included in the cohort if they: (1) were older than 60 years of age, (2) had a unilateral traumatic closed fracture of the upper section of the femoral shaft, with total displacement, (3) had a 32.A or 32.B fracture pattern, according to the AO/OTA classification[7], (4) were to be treated by closed reduction and anterograde interlocking intermedullary nail, and (5) underwent operation within 7 days from injury. Patients were excluded from the study if they had: (1) an open fracture, (2) multiple fractures, (3) pathologic fracture, or (4) declined to participate in this cohort. 92 patients with femoral shaft fracture were considered for inclusion in this trial. Of these, 14 failed to meet the inclusion criteria, and ten declined to participate in the trial (Fig 1).

### 2 Structure of the instrument

The instrument can be divided into 2 components: a fracture reduction device and a guidewire-aiming device.

#### 2.1 Fracture reduction device

**Fig 2:**
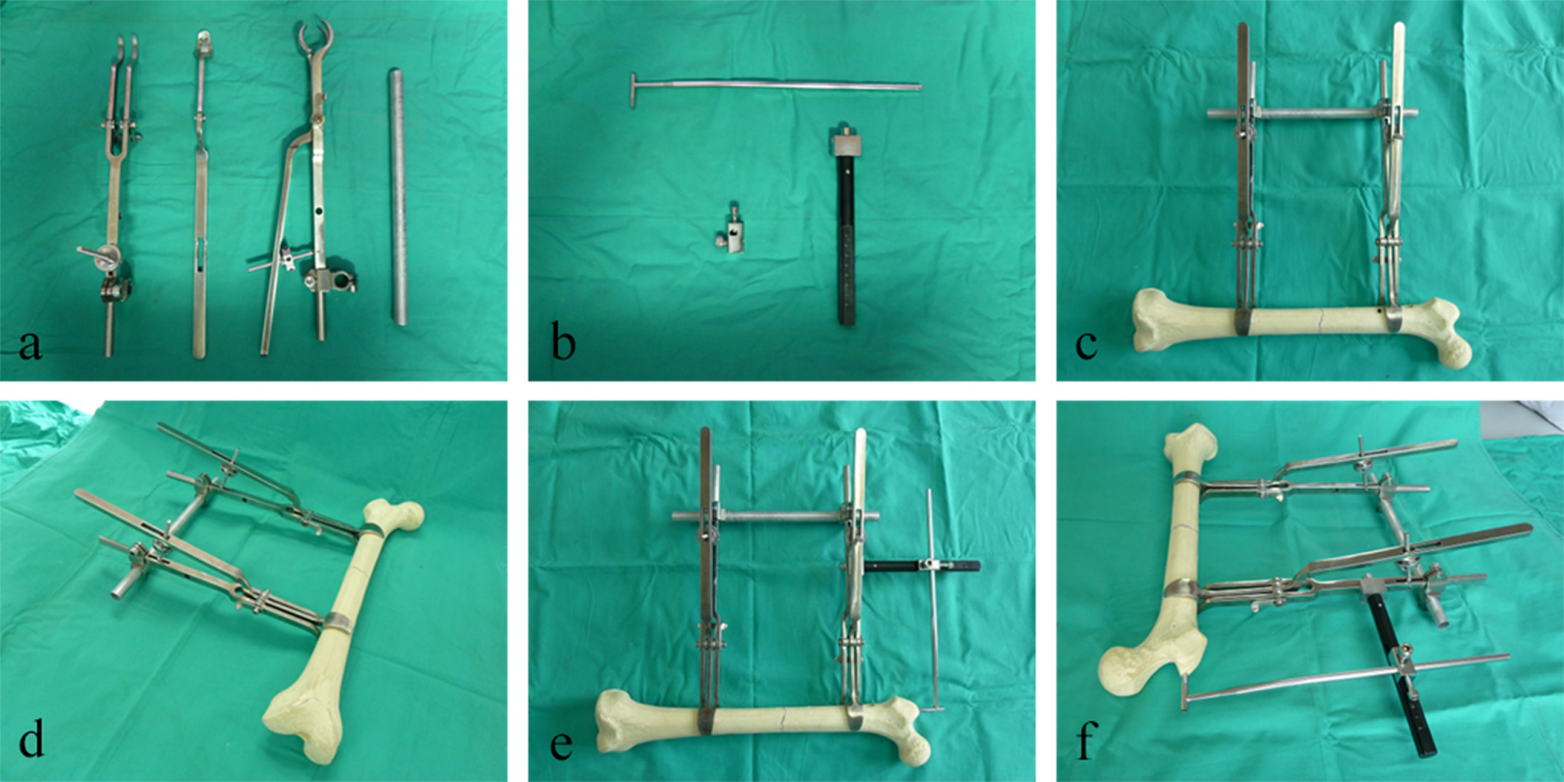
Picture of the instrument developed. (a) bone forceps, bar, and connectors of the fracture reduction device; (b) column, calibrated bar, and slider of the guidewire-aiming device; (c, d) assembled fracture reduction device on a model of the femur; (e, f) assembled fracture reduction device and guidewire-aiming device on a model of the femur.

The fracture reduction device consists of 2 bone forceps, 1 bar, and 2 connectors (Fig 2A). Each set of bone forceps consists of 2 tong arms, connected to each other with a lock catch. Thus, the opening angle of the forceps is detachable and adjustable. The 2 tong jaws of the bone forceps are different; 1 has a double row of narrow teeth, and the other a single row of wide teeth, which will enhance the grasping force. There is a hole near the end of the tong arm for insertion of the aiming frame of the guidewire-aiming device. In addition to the forceps, the instrument includes a bar, which is a hollow circular tube. Finally, a connector exists, composed of 2 mutually perpendicular holes, in perpendicular planes, which separately match the size of the bone forceps and the bar. The bone forceps and the bar can be locked through the connector using screws.

#### 2.2 Guidewire-aiming device

The guidewire-aiming device consists of a column, calibrated bar, and a slider (Fig 2B). One end of the column component matches the hole in the lateral side of the tong arm of the bone forceps, and can be locked using a screw. The calibrated bar is a tube with a scale, at the end of which there is a short sleeve. Finally, the slider is able to move freely along the column, and can be locked using a screw. There is a hole in the slider matching the cross-section of the calibrated bar, which may also be locked with a screw.

### 3 General materials

The protocol of this trial and supporting CONSORT checklist are available as supporting information; see S1 CONSORT Checklist and S1 Protocol. From February 2011 to December 2013, all patients admitted to our department with a diagnosis of femoral shaft fracture were considered for this study. All femoral shaft fractures were categorized according to the AO/OTA classification by at least 2 surgeons. Enrolled patients were randomly allocated to one of the treatment groups, with or without the novel instrument to be used in the operative process, using sealed, opaque envelopes. Each envelope contained a card, indicating the treatment for each patient. Each participant had a follow-up period of at least 1 year to assess the progress of fracture healing. Patients were followed up clinically and radiologically at 1 day, and 1, 3, 6, 9 and 12 months, postoperatively.

### 4 Operative techniques

After general anesthesia, the patient was placed on a traction bed in the supine position. The shortening displacement of the limb was corrected by traction. A 3 cm long incision was made on the lateral side of the thigh, at approximately the position of the lesser trochanter. One tong arm of the bone forceps was placed in the incision to grip the posterior portion of the femur, while the other was placed in the incision to grip the anterior portion. Then, the tong arms were assembled together as forceps using a lock catch. Next, the bone forceps were adjusted to be vertical with the femur. The other set of bone forceps were assembled and applied on the lower edge of the fracture, following the same procedure. Two connectors were attached to the posterior tong arms of the 2 bone forceps, and then locked when the connectors were adjusted to be at equivalent locations on both tong arms. The bar was placed through the holes in each of the 2 connectors. The distal bone forceps were then adjusted to be in the same plane with the proximal, and the screws tightened to lock the connector as a means of avoiding mutual movement between the bone forceps and the bar. The quality of closed reduction was examined using fluoroscopy, and any required adjustments were made.

**Fig 3:**
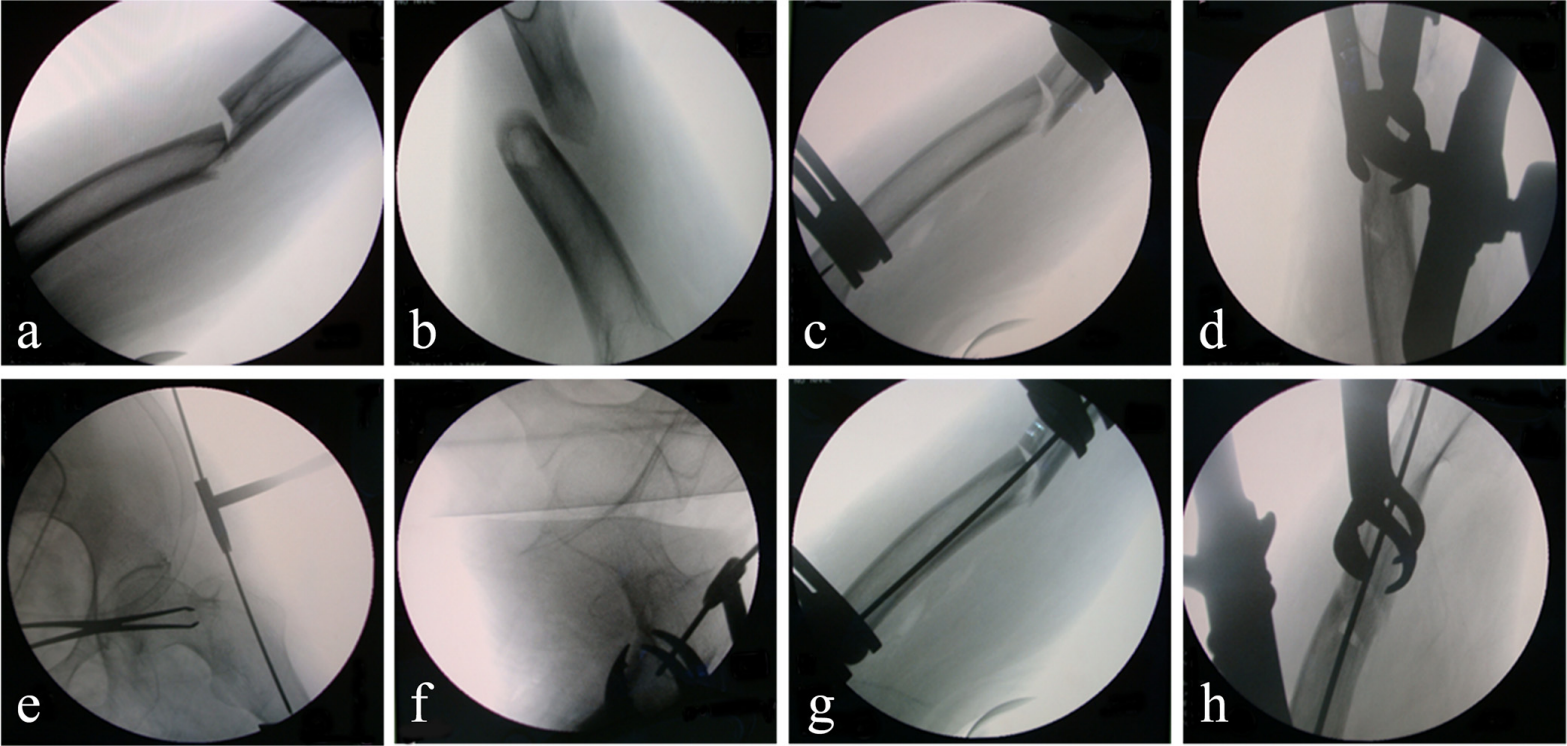
Radiographs showing application of the instrument in an operation for a femoral shaft fracture. (a, b) preoperative condition of the fracture (c, d) completed closed reduction with fracture reduction device (e, f) drilling into the proximal femoral medullary cavity using a Kirschner wire (1.5 mm) through the short sleeve (g, h) drilling into the distal femoral medullary cavity using a Kirschner wire.

After the completion of fracture reduction, the column with a slider on it was connected to the proximal bone forceps through the hole on the lateral side of the posterior tong arm, and subsequently tightened. The calibrated bar was then placed through the hole in the slider and adjusted to make the short sleeve at the end of it touch the hip; located approximately above the greater trochanter. A screw was tightened to temporarily fix the connection between the calibrated bar and slider. A 4 cm long incision was made under the short sleeve. The aiming frame screws were undone and the calibrated bar adjusted to make the ‘scale 4’ overlap with the inside edge of the slider, after which the screw was tightened again to fix the frame. A guidewire was drilled into the proximal femoral medullary cavity through the short sleeve. The aiming frame was then removed. The entrance was expanded using a hollow drill. The guidewire was then drilled into the medullary cavity, extending all the way to the distal fragment. From herein, the procedure was performed in keeping with general intramedullary nail fixation. Fig 3 displays the intraoperative fluoroscopic image of the instrument applied.

Procedures in the control group were performed in accordance with standard operative procedures for intramedullary nailing. The fracture reduction device and guidewire-aiming device were not used in the control group.

Passive exercise was advised 1 week after operation. Mobilization using double crutches, with weight bearing on the uninjured side, was attempted from 4 to 6 weeks, postoperatively.

### 5 Evaluation of outcomes

Data were recorded for all patients, including operative time, operative blood loss, frequency of guidewire drilling, number of open reduction cases, and duration of hospital stay. The incidence of any postoperative complications was recorded.

### 6 Ethics statement

The enrollment of participants begun after this study was approved by the Ethics Committee of Shanghai Sixth People’s Hospital (August 22, 2010). The trial was later registered in ChiCTR (trial registration number ChiCTR-ICR-15007335) as we had neglected to do so. The authors confirm that all ongoing and related trials for this intervention are registered. Patient selection was performed upon admission. Written informed consent was obtained from all individual participants included in the study. This study conforms to the recommendations established by the CONSORT statement (www.consort-statement.org).

### 7 Statistical analysis

Data analysis was performed with SPSS 19.0 statistics software package (SPSS Inc., IBM, Chicago, Illinois). Sex, fracture pattern, and number of open reduction cases were compared using the χ2-test. The frequency of guidewire drilling attempts was compared using the Mann-Whitney *U*-test. Age, preoperative delay, operative time, and operative blood loss were compared with Student’s *t*-tests. Statistical significance was assumed at p < 0.05.

## Results

Injuries of the participants were caused by road traffic and falling from height. Sixty-eight eligible patients were randomized into the 2 treatment groups. The patients enrolled in different groups were similar with regard to age, sex, affected side, and preoperative delay. The fracture patterns in both groups did not differ significantly with application of AO/OTA classification (Table 1).

**Table 1:** Details of all patients, in both groups, with femoral shaft fracture.

	Experimental group	Control group
**Sex (Male/Female)**	23/11	20/14
**Age (years)**	37.32 ± 8.40	35.12 ± 9.34
**Injured side (Left/Right)**	20/14	18/16
**AO/OTA classification (32.A/32.B)**	14/20	16/18
**Preoperative delay (days)**	4.38 ± 1.46	4.29 ± 1.47

The operative time was significantly shorter for the experimental group at 72.71 ± 6.83 minutes, compared with 81.47 ± 6.11 minutes for the control group (p < 0.01). The frequency of drilling and the number of open reduction cases in the experimental group were significantly smaller than those in the control group (p < 0.01). The calculated operative blood loss was slightly less for the experimental group, but did not exhibit significance (p = 0.123) (Table 2). There was no significant difference for the length of hospitalization between the groups. None of the patients, in either group, required a transfusion after operation. No clinically-severe limitations in hip or knee movements were observed in patients of either group.

**Table 2:** Comparison of the operative time, operative blood loss, frequency of drilling, number of open reduction cases, and hospitalization time between groups.

	Experimental group	Control group	p value
**Operative time (min)**	72.71 ± 6.83	81.47 ± 6.11	< 0.001
**Operative blood loss (mL)**	102.65 ± 16.75	109.21 ± 17.81	0.123
**Frequency of drilling (median)**	1	3	< 0.001
**Number of open reduction cases**	0	4	0.042
**Hospitalization (days)**	6.32 ± 1.37	6.41 ± 1.4	0.794

All patients successfully obtained fracture union, with the exception of 1 case of non-union in the experimental group and 2 in the control group. Patients with non-union obtained bone union with fracture clearing and autologous iliac bone grafts. Patients in both groups recovered well with respect to limb function, with no nail loosening or rupture. Superficial infection affected the recovery of 2 patients in the control group who had undergone additional incision for fracture reduction, due to the failure of closed reduction. Infections were treated using oral broad-spectrum antibiotics and a daily dressing change for the sites involved. Long-term influences were not observed in the patients with superficial infection.

## Discussion

Closed reduction and antegrade intramedullary nailing internal fixation has become the standard method of treatment for femoral shaft fracture of the upper or middle segment [6,8–10]. However, surgeons must overcome 2 major difficulties during the operation. One of these is the difficulty of closed reduction. When femoral shaft fracture occurs, the femur exhibits shortening, and angular, lateral, or rotational displacement resulting from muscle compressive effects[11–14]. Shortening, angular, or rotational displacement can be corrected using a traction table. However, there remains no effective instrument to assist in the reduction of lateral displacement. In most cases, closed reductions are performed by hand. Due to the thick thigh muscle and deep fracture location, reduction often cannot be completed and maintained adequately using just the hands or simple instruments. Further, it is difficult to insert a guidewire into the medullary cavity from the peak of the greater trochanter, a procedure that is complicated by difficulty in determining the point of entry. The entry point for the intramedullary nail is located on the medial side of the peak of the greater trochanter. In the conventional and physiological anatomic arrangement, the gluteus medius and gluteus minimus are attached around the peak of the greater trochanter, and the entry point is located on a diagonal bone surface extending from the femoral neck, making the entry point area very small and difficult to drill. Additionally, the proximal fracture fragments typically shift laterally, and thus the greater trochanter tends to shift deeper within the body, resulting in a larger angle between the longitudinal axis of the proximal fragment and the drilling direction, making the guidewire insertion much more difficult. Inserting the guidewire into the medullary cavity accurately from the peak of the greater trochanter plays a key role in the operation, after successful reduction of the fracture[2,15–17]. Currently, surgeons without extensive surgical experience often require multiple rounds of drilling and fluoroscopy, which can cause iatrogenic injury and increase the risk of infection. A simple reduction clamp application can assist in fixation and improve operative quality to some extent[18–21], but difficulties persist in maintaining the reduction and accurate determination of an entry point.

To resolve these problems, we designed and manufactured an instrument to be used in treating femoral shaft fractures. The principle of the fracture reduction device is as follows. The bone forceps, bar, and femoral shaft form a quadrilateral structure when assembled as a complete instrument. Given that right angles exist between the bone forceps and femoral shaft, and between the bone forceps and bar, any lateral displacement is corrected when the bar passes through the 2 bone forceps at equivalent locations. Similarly, the anterior-posterior displacement is corrected when both bone forceps and the bar are in the same plane. In the final arrangement, the instrument is fixated in approximately a rectangular arrangement, with the angular and lateral displacement corrected. Reduction is maintained with solid fixation of the device (Fig 2C and 2D).

The principle of the guidewire-aiming device is as follows. The central annular area of the head of the bone forceps is positioned at almost the center of medullary cavity, when the proximal fragment is clamped vertically by the bone forceps. Thus, the central annular area of the head of the bone forceps is located approximately along the central axis of femur. The aiming device is L-shaped, one end of which is connected to the tong arm of the proximal bone forceps, with the other end having a short sleeve. In the preparation stage, the L-shaped aiming device is connected to the tong arm of the bone forceps, with the calibrated bar then adjusted to ensure that the short sleeve is positioned aiming toward the center of the annular area of the head of the bone forceps. The relative location of the slider on the calibrated bar was recorded (the medial edge of the slider overlaps with the ‘scale 4’ of the calibrated bar) (Fig 2E and 2F). On this basis, the short sleeve aims accurately at the peak of the greater trochanter during the operation. It was also found that the entry point is more accurate when the proximal bone forceps are closer to the lesser trochanter and more vertical to the femoral shaft.

In this study, the frequency of drilling into the medullary cavity and the number of open reduction cases were significantly smaller in the experimental group than those in the control group, indicating that this instrument plays an important role in determining the entry point and maintenance of fracture reduction. In the experimental group, 27 cases had successful drilling into the proximal medullary cavity on the first attempt. Another 7 cases did not exhibit successful drilling into the medullary cavity on the first attempt, resulting from an inaccurate entry point, which is believed to be a product of the proximal bone forceps being insufficiently vertical with the femoral shaft. All 7 cases of these cases had successful drilling on the second attempt, after instrument readjustment. Generally, the drilling in the control group required at least 3 attempts, and was as high as 6 attempts in 2 cases, which increases unnecessary operative time and may cause iatrogenic injury. With the solid fixation of the fracture reduction device, the guidewire could be successfully inserted into the distal medullary cavity in the first attempt. Therefore, no case in the experimental group required open reduction. However, in the control group, 4 patients required open reduction, as the guidewires were unable to reach the distal medullary cavity with unstable fracture reduction. As previously established, exposing the fracture site can impair the periosteal blood supply and thus affect the healing of the fracture [22].

The application of this instrument would theoretically be accompanied by an increase in operative time. In our study, however, operative time in the experimental group was less than that of the control group (p < 0.05). We suggest that this may be a product of our instrument being simple to operate and convenient to assemble in a short time. Additionally, the improvement in reduction quality and efficiency of guidewire drilling may also contribute to a reduced operative time.

The number of incisions performed was greater in the experimental group, due to the use of the instrument. As the results demonstrate, the operative blood loss in the experimental group was more than that of the control group, but no statistical significance was observed between the groups (p > 0.05). This lack of significance may be attributable to fewer guidewire drilling attempts and a shorter operative time in the experimental group.

No statistical significance regarding hospitalization time or union rate was observed between the groups (p>0.05), indicating that this instrument has little effect on postoperative recovery.

## Conclusion

Femoral shaft fractures can be easily reduced and temporarily fixated using the novel fracture reduction device without exposing the fracture area. Further, the entry point can be quickly determined using the guidewire-aiming device. The guidewire can be drilled into the distal medullary cavity from the entry point with ease, following application of the instrument, establishing a good foundation for subsequent procedural steps. The instrument is simple in structure, and easy to operate. The learning curve for using of the instrument is short. The application of the instrument can greatly reduce dependence on the operative experience of the surgeon, and may offer substantial assistance to newer orthopedic surgeons. Appropriate utilization of this novel instrument can effectively improve the quality of operation and reduce harmful X-ray exposure.

## Supporting Information

S1 CONSORT Checklist (DOC)

S1 Database (XLSX)

S1 Protocol (DOCX)
