# Supplementary material for: Improve the Efficiency of Surgery for Femoral Shaft Fractures with A Novel Instrument: A Randomized Controlled Trial
Source: PLoS One. 2016 Apr 26;11(4):e0154332. doi: 10.1371/journal.pone.0154332 (PMC4846024; doi:10.1371/journal.pone.0154332)
Supplement: S1 Protocol — (DOCX) [file pone.0154332.s003.docx]

**新式手术器械在股骨干骨折手术的应用研究：随机对照试验**

**背景**

世界范围内青年人的死亡40%是由创伤引起。股骨干骨折是创伤骨科常见的骨折之一，约占非致命道路交通损伤的9%。股骨干骨折的死亡率约为0.04%[1]。近年来，对股骨干骨折治疗的研究是当下创伤骨科领域的一大热点。

交锁髓内钉复位内固定用于股骨干骨折的治疗符合生物学固定理念，且有手术创伤小、术中出血少、术后骨折愈合率高等优点，目前已成为股骨干骨折最常用的手术治疗方法[2, 3]。其手术方式可分为切开复位和闭合复位两种。切开复位可使术者在直视下操作，使骨折达到良好的复位，但由于切开复位的过程会破坏骨折端局部的血运，使手术失血量增大，感染机会明显增加，且有更高的骨折延迟愈合和骨折不愈合的风险。大量临床实践表明闭合复位相较切开复位虽然不一定能达到骨折端的解剖复位，但由于它对软组织损伤轻、不破坏骨折端血液循环，可明显减少失血和感染率。同时，其骨折愈合的时间明显短于切开复位，愈合强度也较好。

对股骨干骨折而言，顺行交锁髓内钉闭合复位内固定的常规手术过程中需要从大转子顶点将导针顺行插入股骨近端的髓腔，并通过骨折端进入骨折远端的髓腔，在这个过程中会遇到两大难题。

第一个难题在于如何有效的维持骨折的复位。由于大腿部肌肉丰富，收缩力量强大，骨折后骨折断端在肌肉牵拉下常造成严重移位。对于短缩畸形和成角畸形，术中可通过牵引床行足部纵向牵引来纠正，而对骨折的侧方移位，则只能通过手法复位来解决。目前尚无一种有效的复位器来辅助复位，大多数情况下只能通过手法牵引复位来完成。由于大腿肌肉厚，骨折端位置深，单纯依靠徒手操作常常无法完成和维持复位。首先，严重移位的骨折在复位过程中，纠正左右侧方移位后再纠正前后移位时无法有效维持左右方向的对位。其次，徒手操作无法与丰富的大腿肌肉进行长时间的对抗，在用C臂X光机透视确认骨折端是否有复位时以及在大转子顶点进针和插入导针的过程中骨折端均有可能发生移位。骨折复位不良不仅影响髓内钉的植入，同时也会增加骨折畸形愈合、骨折延迟愈合和骨折不愈合的风险。另外，有临床证据表明，即使是股骨轻微的旋转畸形也会明显改变膝关节的接触力，使骨关节炎的发病率增加[4]。

第二个难题在于如何有效确保髓内钉导针自大转子顶点插入并顺利进入骨折断端的远端髓腔。首先，导针进针点的确定有一定的难度。髓内钉的插入点一般选择大转子顶点前中三分之一区域交界处略偏内的位置，由于大转子顶点有臀中小肌附着，且其内侧很快以陡坡延续至股骨颈，因此可供进针的面积很小，且不容易确定。即使确定了进针点，在进针过程中针尖也易移位。不正确的进针点可导致髓内钉植入困难，甚至可造成医源性损伤。其次，近侧骨折端由于在肌肉牵引及会阴部对抗牵引柱的作用下，常向外上方移位，从而使大转子顶点更向身体深部倾斜，使进针方向与骨折近侧段纵轴的夹角变小，造成进针困难。

近几年来，计算机导航系统逐渐发展，并应用于股骨干骨折的治疗中[5]。其主要原理在于利用从健侧和患侧的近端及远端收集的X线影像，计算出股骨的长度、股骨颈前倾角以及旋转轮廓等，通过匹配患肢与健肢的长度和旋转度，指导髓内钉的植入和锁定。但计算机导航系统的使用仍无法解决股骨骨折闭合复位困难的问题，而且计算机导航系统往往受到照相机系统的有效视野限制，术中常需要多次调节照相机的位置，这将明显增加手术时间。另外，此项技术操作复杂，手术仪器费用昂贵。无法有效推广。

目前，股骨干骨折的闭合复位和髓内钉进针点及进针方向的确定主要依赖于术者的经验及C形臂X光机的透视。因此若能设计一种具有辅助骨折闭合复位及进针瞄准功能的器械将使手术变得简单易行。

**研究目的**

我们拟设计并制造一种操作简便又能有效辅助手术的股骨干骨折复位器和髓内钉进针瞄准器。通过临床应用评价股骨干骨折复位器和髓内钉进针瞄准器的实用性与有效性，探讨其临床应用价值和应用前景。

**研究设计**

前瞻性随机对照试验

研究对象来源：上海市第六人民医院骨科

**入组病例数**

68例

**入组标准**

（1）患者年龄不大于60岁。

（2）外伤性单侧股骨干中上段闭合性骨折，骨折端完全移位。

（3）AO/OTA分型为32.A及32.B型骨折

（4）拟闭合复位后使用顺行交锁髓内钉内固定。

（5）受伤至手术时间短于7天。

**排除标准**

（1）开放性骨折。

（2）多发性骨折。

（3）病理性骨折。

（4）拒绝参与本研究。

**干预措施**

实验组和对照组均完善相关术前检查，排除手术禁忌，签署手术同意书。试验组的主要手术步骤为首先使用牵引床与股骨干骨折复位器进行骨折的复位。复位满意后，安装髓内钉进针瞄准器定位导针的穿针的位置。沿导针套管打入髓内钉导针，直至穿过骨折远端髓腔。拆除复位器和瞄准器，继续植入髓内钉。对照组在术中不得使用我课题组设计的股骨干骨折闭合复位器和髓内钉进针瞄准器，其余主要手术步骤和试验组无明显差别。即主要依靠牵引床和手法牵引复位骨折，然后凭借术者的经验和手感从大转子顶端打入导针，借助C臂X光机的透视将导针穿入骨折远端。之后按照标准方法植入髓内钉和近端及远端螺钉。

**评价指标**

本临床研究的评价指标包括手术时间、术中出血量、导针穿针次数、切开复位的例数、住院时间、术后并发症、骨折愈合情况。

手术时间是指手术中第一次切开皮肤时开始，直至所有切口缝合完毕所需要的时间。包括手术入路、术中复位、术中透视、装卸股骨干骨折闭合复位器、装卸髓内钉进针瞄准器、植入导针和髓内钉、植入远近端螺钉及关闭切口的时间。术中出血量是指手术时间以内的出血量。导针穿针次数是指导针从大转子顶点朝正确方向穿入股骨近端髓腔所需要的穿针总次数，即穿针次数至少为1次。切开复位的例数具体是指因骨折闭合复位效果不佳导致多次穿针仍无法将导针准确穿入骨折远端髓腔，不得已行切开复位的病例数。术后并发症包括但不限于浅表感染、髓内钉松动断裂、髋膝关节活动受限等。术后1天及1、3、6、9、12个月时随访并行X线片检查，观察骨折愈合情况。骨折术后9个月骨折未达到临床愈合标准可诊断为骨不连。

**统计学分析**

应用SPSS 19.0统计学软件，采用χ^2^检验分析两组患者的性别、骨折分型、切开复位的例数是否有统计学差异；采用两独立样本秩和检验比较两组患者的导针穿针次数是否有统计学差异；采用独立样本t检验分析两组患者的年龄、受伤至手术时间、手术时间、术中出血量、住院时间是否有统计学差异。P＜0.05时认为两组之间的差异具有统计学意义，P≥0.05时认为两组之间的差异无统计学意义。

**签字页**

盛加根

上海市第六人民医院

宜山路600号

徐海涛

上海市第六人民医院

宜山路600号

殷文靖

上海市第六人民医院

宜山路600号

徐佩君

上海市第六人民医院

宜山路600号

秦晖

上海市第六人民医院

宜山路600号

安智全

上海市第六人民医院

宜山路600号

张长青

上海市第六人民医院

宜山路600号

**Improve the Efficiency of Surgery for Femoral Shaft Fractures with A Novel Instrument: A Randomized Controlled Trial**

**Summary in English**

**Background**

Trauma is a neglected source of morbidity and mortality which accounts for 40% of deaths among young people world-wide. Femoral shaft fracture is of particular interest as it accounts for up to 9% of all non-fatal road traffic injuries. Treatment is equally as significant as prevention to reduce trauma-associated disability. Although femoral shaft fracture is commonly not fatal, with an acute mortality rate of approximately 0.04%[1], such fractures would cause substantial mortality and morbidity if treated without benefit of traction or reduction. Options for treatment of femoral fractures have become increasingly available and proven to be effective.

In recent years, Closed reduction and interlocking intramedullary nail internal fixation has become the standard treatment for femoral shaft fracture[2, 3]. However, there are two difficulties during this operation. First of all, it is not easy to determine the entry point and direction of guidewire for drilling into the proximal medullary cavity. Moreover, there is also difficulty for temporary fixation and maintenance of fracture reduction to make it easy for drilling guidewire into distal medullary cavity. Currently, there is still no convenient and effective instrument assisting to resolve these two problems.

**Purpose**

To design an instrument that could assist in closed reduction and guidewire aiming during the intramedullary nail internal fixation for femoral shaft fractures, and to validate its efficacy via clinical appliance.

**Study design**

Prospective, randomized, controlled study.

Setting: Department of Orthopedics, Shanghai Sixth People’s Hospital

**Study population**

68 patients

**Inclusion criteria**

Patients were included in the cohort if they (1) were not beyond 60 years old, (2) had a unilateral traumatic closed fracture of the upper section of the femoral shaft, which was totally displaced, (3) were 32.A or 32.B fracture pattern according to the AO/OTA classification, (4) were to be treated by closed reduction and anterograde interlocking intermedullary nail, (5) underwent operation in 7d from the injury.

**Exclusion criteria**

Patients were excluded from the study if they had (1) open fracture, (2) multiple fractures, (3) pathologic fracture, (4) declined to participate in this cohort.

**Interventions**

The novel instrument was used over the operation in experimental group for fracture reduction and guidewire aiming while it was not used in control group.

**Outcomes evaluation**

The data measured for all patients included operative time, operative blood loss, frequency of guidewire drilling, case number of open reduction, duration of hospital stay. The incidence of any postoperative complications was recorded. Patients were followed up clinically and radiologically at 1 day, 1, 3, 6, 9 and 12 months to determine the fracture healing situation.

**Statistical analysis**

The data were analyzed by SPSS 19.0 statistics software package (SPSS Inc, IBM, Chicago, Illinois). Gender, fracture pattern and case number of open reduction were compared by χ2-test. Frequency of guidewire drilling attempts was compared by Mann-Whitney *U*-test. Age, preoperative delay, operative time, operative blood loss were compared by Student’s *t*-test. Statistical significance was considered when p < 0.05.

**Study flowchart**

Candidate enrolment

Treatment without the novel instrument used in the operation

Treatment with the novel instrument used in the operation

Outcomes measurement

Follow-up for fracture healing

**References**

1. Baker SP, O'Neill B, Haddon W, Jr., Long WB. The injury severity score: a method for describing patients with multiple injuries and evaluating emergency care. The Journal of trauma. 1974;14(3):187-96. PubMed PMID: 4814394.

2. Lin SJ, Chen CL, Peng KT, Hsu WH. Effect of fragmentary displacement and morphology in the treatment of comminuted femoral shaft fractures with an intramedullary nail. Injury. 2014;45(4):752-6. Epub 2013/11/26. doi: 10.1016/j.injury.2013.10.015. PubMed PMID: 24268188.

3. Sekimpi P, Okike K, Zirkle L, Jawa A. Femoral fracture fixation in developing countries: an evaluation of the Surgical Implant Generation Network (SIGN) intramedullary nail. The Journal of bone and joint surgery American volume. 2011;93(19):1811-8. Epub 2011/10/19. doi: 10.2106/jbjs.j.01322. PubMed PMID: 22005867.

4. Bretin P, O'Loughlin PF, Suero EM, Kendoff D, Ostermeier S, Hufner T, et al. Influence of femoral malrotation on knee joint alignment and intra-articular contract pressures. Arch Orthop Trauma Surg. 2011;131(8):1115-20. Epub 2010/11/12. doi: 10.1007/s00402-010-1210-4. PubMed PMID: 21069363.

5. Khoury A, Liebergall M, Weil Y, Mosheiff R. Computerized fluoroscopic-based navigation-assisted intramedullary nailing. Am J Orthop (Belle Mead NJ). 2007;36(11):582-5. Epub 2007/12/14. PubMed PMID: 18075604.
